# Discovery of Genome-Wide Microsatellite Markers in *Scombridae*: A Pilot Study on Albacore Tuna

**DOI:** 10.1371/journal.pone.0141830

**Published:** 2015-11-06

**Authors:** Natacha Nikolic, Stéphanie Duthoy, Antoine Destombes, Nathalie Bodin, Wendy West, Alexis Puech, Jérôme Bourjea

**Affiliations:** 1 IFREMER, Institut Français de Recherche pour l’Exploitation de la Mer, Délégation de La Réunion, Rue Jean Bertho, BP 60, 97 822 Le Port Cedex, La Réunion, France; 2 GENOSCREEN, Rue du Professeur Calmette, 59000 Lille, France; 3 IRD, UMR 212 EME, BP 570 Victoria, Mahé, Seychelles; 4 Department of Agriculture Forestry and Fisheries, Private Bag X2, Roggebaai, 8012, South Africa; Chinese Academy of Fishery Sciences, CHINA

## Abstract

Recent developments in sequencing technologies and bioinformatics analysis provide a greater amount of DNA sequencing reads at a low cost. Microsatellites are the markers of choice for a variety of population genetic studies, and high quality markers can be discovered in non-model organisms, such as tuna, with these recent developments. Here, we use a high-throughput method to isolate microsatellite markers in albacore tuna, *Thunnus alalunga*, based on coupling multiplex enrichment and next-generation sequencing on 454 GS-FLX Titanium pyrosequencing. The crucial minimum number of polymorphic markers to infer evolutionary and ecological processes for this species has been described for the first time. We provide 1670 microsatellite design primer pairs, and technical and molecular genetics selection resulting in 43 polymorphic microsatellite markers. On this panel, we characterized 34 random and selectively neutral markers («neutral») and 9 «non-neutral» markers. The variability of «neutral» markers was screened with 136 individuals of albacore tuna from southwest Indian Ocean (42), northwest Indian Ocean (31), South Africa (31), and southeast Atlantic Ocean (32). Power analysis demonstrated that the panel of genetic markers can be applied in diversity and population genetics studies. Global genetic diversity for albacore was high with a mean number of alleles at 16.94; observed heterozygosity 66% and expected heterozygosity 77%. The number of individuals was insufficient to provide accurate results on differentiation. Of the 9 «non-neutral» markers, 3 were linked to a sequence of known function. The one is located to a sequence having an immunity function (ThuAla-Tcell-01) and the other to a sequence having energy allocation function (ThuAla-Hki-01). These two markers were genotyped on the 136 individuals and presented different diversity levels. ThuAla-Tcell-01 has a high number of alleles (20), heterozygosity (87–90%), and assignment index. ThuAla-Hki-01 has a lower number of alleles (9), low heterozygosity (24–27%), low assignment index and significant inbreeding. Finally, the 34 «neutral» and 3 «non-neutral» microsatellites markers were tested on four economically important Scombridae species—*Thunnus albacares*, *Thunnus thynnus*, *Thunnus obesus*, *and Acanthocybium solandri*.

## Introduction

Albacore tuna (*Thunnus alalunga*) is a highly migratory tuna species found in both subtropical and temperate waters of the three oceans and in the Mediterranean [1]. With a high commercial value [2], this species is mainly targeted by pelagic fisheries in all ocean basins and current catches are estimated to represent 5% of the global tuna catch [2, 3]. As such, it is the responsibility of regional fisheries management organizations, such as the Indian Ocean Tuna Commission (IOTC), to oversee the management and sustainable harvesting of this species. Several stocks of albacore are currently considered fully exploited or overexploited, although considerable uncertainty remains in the results of stock assessment due to fisheries statistics and species biology uncertainties (e.g. for the Indian Ocean; [4]). Therefore precautionary approach to the management of albacore should be applied and it remains a priority to improve stock assessments of this species, through the development of alternative methods of population assessment [5, 6, 7].

Scientific results are the baseline to improve the management of a species and investigation of population structure provides key information to improve stock assessments [8]. The stock structure assumed during an assessment process has important consequences in the management and must be as close as possible to the actual population structure of the resource [9]. Population genetics have much to offer to improve stock structure for fisheries management. For example, whereas all tuna species are highly migratory, genetic differentiation has been detected at various scales, within an ocean basin for bluefin tuna *Thunnus thynnus* [10], and both within and among oceans for the yellowfin tuna *Thunnus albacares* [11] and bigeye tuna *Thunnus obesus* [12, 13]. Information on the population structure of albacore and its habitats are unfortunately scarce (see review of albacore stock structure in [14]. For instance, the Indian Ocean is the oceanic region in which the least knowledge of albacore is available and, in lieu of the results of recent albacore stock assessments, the IOTC Scientific Committee has encouraged studies on the population structure within the Indian Ocean and adjacent waters [15, 4].

Over the past several years, mainly by using 454 pyrosequencing, genome-wide microsatellite screening and marker development has been performed in many non-model species, such as fish, for genetic and molecular ecology study [16, 17, 18, 19]. Next-generation sequencing technology (454) with the reduced representation library (RRL) construction rapidly and easily isolates the microsatellite of the genome of the non-model teleost at low cost and time [19]. In this study, we used the high throughput 454 technology from an enriched microsatellites library on albacore tuna to insulate rapidly, easily and flexibly microsatellite on the whole genome.

Genetic markers are widely used to investigate genetic diversity within populations, connectivity between populations, and to identify stocks and mixed stocks in a fishery [20, 21]. Molecular genetics has led to considerable progress but to unravel population structures, studies are dependent on the use of polymorphic neutral markers. Neutral markers usually indicate a DNA region that is not under the influence of selection, and the vast majority of genetic diversity estimates are based on neutral markers [22]. Neutral markers that are capable of inferring genetic diversity are most commonly microsatellites [22]. The hypothetically random and selectively «neutral» markers are mentioned in this study. Microsatellites markers have much to offer in fisheries management (see the review in [23, 24]). These genetic markers are used in a variety of population genetic studies on marine species because of their high locus variability allowing high statistical power to detect genetic structure within and among populations, as well as inferring evolutionary history [25, 26, 27, 28, 29]. Due to their cosmopolitan distribution, large population size, high fecundity, production of numerous pelagic larvae, long larval periods allowing widespread dispersal in currents and due to the ability of adults to easily migrate inter-ocean distances [30]; marine pelagic fish species have commonly been thought to lack genetic spatial structure [31, 32]. In this last decade, genetic studies using microsatellites in pelagic fish investigations have increased [33, 34, 35, 36, 37, 38, 39, 40, 41, 42, 43, 44, 45]. Microsatellites have been characterized from *Thunnus thynnus*, *Thunnus orientalis*, *Thunnus obesus*, *Thunnus albacares*, yet none have been specifically designed for albacore tuna. Some of the markers developed on bluefin ([46], [47] (4 markers), [48] (24 markers)) were tested on albacore to study the population structure of albacore in the Atlantic ([42] (12 markers), [44] (13 markers)). These studies revealed contrasting results and have fuelled the need for an increase in the number microsatellite markers to be able to spatial structure in such pelagic species. In this short communication, we describe the development of new appropriate microsatellite markers for extensive population genetic analysis on albacore using shotgun pyrosequencing of a microsatellite-enriched library [49], and the power analysis. Additionally, these new microsatellites markers have been tested with four other Scombridae species (*Thunnus albacares*, *Thunnus thynnus*, *Thunnus obesus*, and *Acanthocybium solandri*).

## Materials and Methods

### Ethics statement

**Fig 1:**
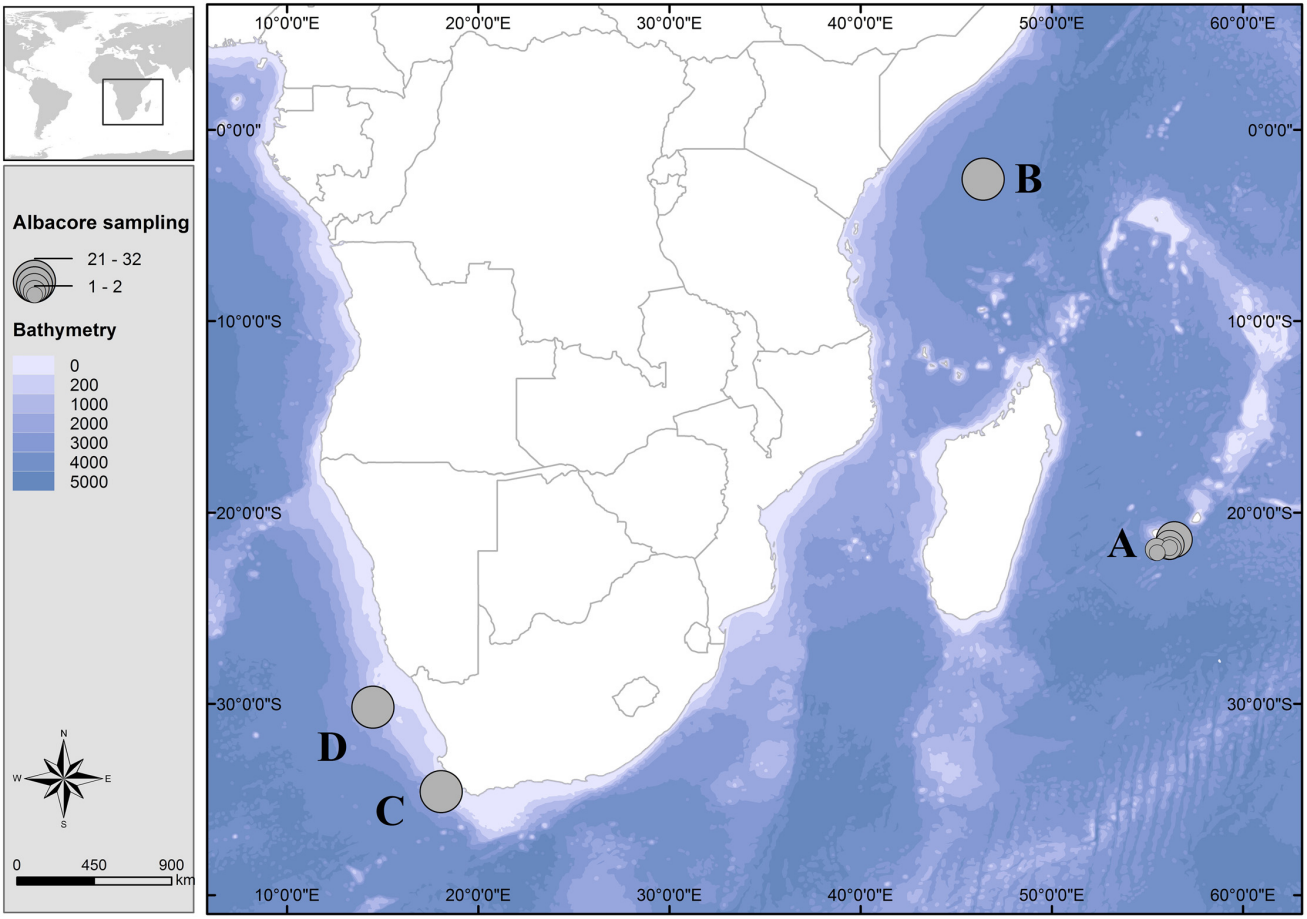
Geographic location of albacore sampled. Circles are proportional to the number of individuals collected. (A) southwest Indian Ocean (n = 42), (B) northwest Indian Ocean (n = 31), (C) South Africa (n = 31), and (D) southeast Atlantic Ocean (n = 32).

The field studies did not involve endangered or protected species. Albacore tuna is a commercial species caught all over the world and does not fall in any official ethical rules (UICN, RED list etc.). No specific permissions were required for the sampling locations (Fig 1). All fishes were randomly sampled from French, Seychelles and South African fishing vessels either at sea within an observer program in the authorized marine waters or at landing sites. The fishing areas are related to the fishing method (mainly longliner and purse seine) and are from one to several kilometers in range.

### Test, procedure, and analysis

Our study includes 136 samples of albacore tuna collected from four different geographic areas, A) southwest Indian Ocean (42), B) northwest Indian Ocean (31), C) South Africa (31), and D) southeast Atlantic Ocean (32) (Fig 1). The Fig 1 was performed using ArcGIS software (www.arcgis.com *)*. Hence, we followed the rule-of-thumb for the estimation of differentiation with > 30 individuals per area [50].

The number of individuals used to develop high quality microsatellite markers in this study varied from 8 to 136 depending on the molecular process. The genomic DNA was isolated from muscle tissue sample (25ng) of a single fish using Qiagen DNeasy spin columns. 1 μg of an equimolar pool of 13 DNA samples was used for the development of a microsatellites library through 454 GS-FLX Titanium pyrosequencing of enriched DNA libraries, as described in [49]. In order to increase the percentage of final sequences with microsatellites, total DNA was enriched for AG, AC, AAC, AAG, AGG, ACG, ACAT, and ATCT repeat motifs and subsequently amplified. Polymerase Chain Reaction (PCR) products were purified, quantified, and GsFLX libraries were then carried out following the manufacturer’s protocols (Roche Diagnostics), and sequenced by 454 GS FLX Titanium pyrosequencing.

A summary of the different selection steps to obtain a final microsatellite panel of markers is presented in Table 1.

**Table 1:** Summary of the selection steps used to develop microsatellite markers.

Steps	Total number	Number of individuals
**Development of sequences**	62 682 sequences with 4 285 microsatellites isolated	13
**Design of microsatellites** (with QDD software)	1670 primer pairs	
**First selection**: best design (for each microsatellite, the closest pair to the optimum parameters of QDD software)	225 primer pairs	
**Second selection**: Tested primer pairs. See S1 Table (ex. motif, length, repeats number, most dinucleotide motif)	95 primer pairs	8
**Third selection**: positive amplification (specific product at expected size for at least 5 samples). See S1 Table	70 primer pairs	
**Fourth selection**: polymorphic study	60 primer pairs	15
**Fifth selection**: technical criteria (genotype readings, weak background, no specific products)	43 markers	
**Sixth selection**: neutral and encoding characteristics	34 neutral and 9 encoding (with two well-known functions) microsatellites	15
**Last selection on neutral markers**: diversity and structure analysis	25 higher quality microsatellites from the 34 markers	136

**Fig 2:**
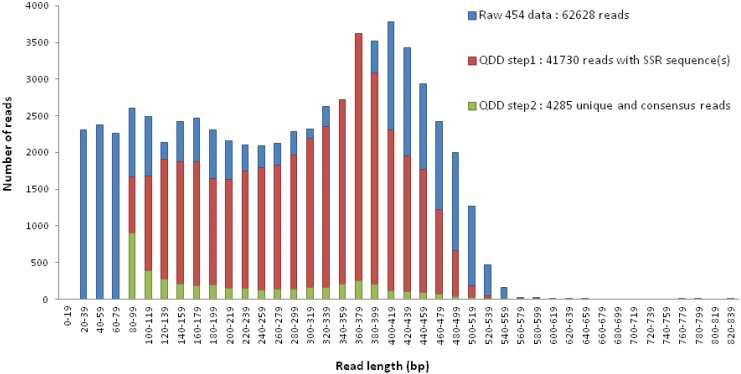
Read length distribution and number of reads throughout QDD bioinformatics pipeline steps.

From the 62 682 sequences obtained, the bioinformatics program QDD [51] was used to filter the primers that designed successfully. This software allowed for high-throughput microsatellite isolation of 4 285 sequences containing SSR motifs, including motifs longer than five repeats (Fig 2).

A total of 1 670 primer pairs were designed (S2 Table for detailed information). Among the 225 microsatellites designed, we retained and tested 95 based on the sequence pattern that would maximize the number of polymorphic markers (S2 Table for detailed information). All primers were tested with one PCR condition in order to apply multiplexed reactions. These consisted of 75 di-nucleotide, 7 tri-nucleotide, 12 tetra-nucleotide, and 1 penta-nucleotide microsatellites primer pairs. Among the 95 candidate loci tested, 25 failed to amplify. From the 60 loci tested for polymorphism, 16 gave inconsistent electrophoretic patterns and 1 showed no or low polymorphism levels. 43 microsatellites markers were interpretable, clear, repeatable, and the polymorphic patterns were validated. Multiplexed loci were built with the same optimal primer pairs annealing temperature of 55°C and can be used for future genetic studies on albacore (see example S1 Fig and Table 2).

**Table 2:** Microsatellite markers developed for Thunnus alalunga (43) with the corresponding GenBank number.

Locus name	Genbank accession number	Sequence Range size (bp)	Primer sequences	Motif	Dye	Blast on the complete sequence >75% (see S2 Table)	Detail on the corresponding alignment (see S2 Table)	Supplement details
ThuAla-mt-01	KM977780	238	F: CAGTGAATGTTTTGCCAACG	(tatc)17	PET			«neutral»
			R: TCATACAGTTTCCCCAAGGC					
ThuAla-Tcell-01	KM977781	203	F: GTCACTGGAGGAACCGGTAA	(gata)17	6FAM	yes	FERM and PDZ domain-containing protein 1-like	«non-neutral»
			R: CCGTGTTGGAGGATCTGAGT					
ThuAla-mt-02	KM977782	302	F: TCAGCAGTCCATCACTTTTCA	(atag)16	PET			«neutral»
			R: TCAAGTCACAGCAGAGATCACA					
ThuAla-mt-03	KM977783	183	F: TGAAGTGCTGGTCTCCAGTG	(gata)15	VIC			«neutral»
			R: TGATTTCTGTTAAGTGGCTGCT					
ThuAla-mt-04	KM977784	133	F: TTCTAAGGAGTGTTGGGTCACA	(tatc)12	NED			«neutral»
			R: CAACATGCAACTACACAAAACA					
ThuAla-Und-01	KM977785	123	F: TCCTTCTCTTCCTCATCTTTCC	(ttct)11	PET	yes	Variant sequence	«non-neutral»
			R: CCACATCTCACTGCCTTCAG					
ThuAla-mt-05	KM977786	218	F: TGGTCTCCCTTGCTGTTACC	(ct)8	6FAM			«neutral»
			R: TCCAGTTCCCACTAGCAACC					
ThuAla-mt-06	KM977787	123	F: CATCATGAAATCCATGCAGC	(ac)9	6FAM			«neutral»
			R: ACATGGTTAACCTGGCGTGT					
ThuAla-mt-07	KM977788	124	F: GCTTCACAAGGCTGGTTACTG	(tg)9	VIC			«neutral»
			R: GGAGGTGGAAACAAGCTCAG					
ThuAla-mt-08	KM977789	134	F: TCTGACCAGTTCAGCTCCCT	(gt)9	PET			«neutral»
			R: TGTTTGCAATGAAATAGTTTTGAA					
ThuAla-mt-09	KM977790	135	F: GCAACCCTTGCTGTCCAATA	(ac)9	6FAM			«neutral»
			R: TCTGTACTGATGAACTCCATGACA					
ThuAla-Und-02	KM977791	144	F: AATGAGGCATTTGCAGCTCT	(gt)9	6FAM	yes	Gene undeterminated	«non-neutral»
			R: CACCGTATTGATCCACTTTGC					
ThuAla-mt-10	KM977792	198	F: CCAGAGATAAATGAATTGAATTAAAGG	(ac)9	PET			«neutral»
			R: CAGCAGCCTTTGCTTTCTCT					
ThuAla-mt-11	KM977793	205	F: TCATGTTCTCACTCGCGTTC	(tc)9	VIC			«neutral»
			R: CCCTTAAACGGGAAGAAACC					
ThuAla-mt-12	KM977794	269	F: TTTCCCTAACATTTGGGCTG	(ac)10	NED			«neutral»
			R: TGGACACAGTGGTGCCTCTA					
ThuAla-mt-13	KM977795	119	F: AGGGAAACGAGGTTCTAGGG	(gt)11	VIC			«neutral»
			R: CCTCCTAATGAGTCCGGAGA					
ThuAla-mt-14	KM977796	125	F: CATGAAGAATAGAATAGCAGCTTTG	(tg)11	NED			«neutral»
			R: TCTGTGAATGGAGACGTTGG					
ThuAla-mt-15	KM977797	149	F: GATTGCGCAACAATCAAAGA	(tc)11	VIC			«neutral»
			R: GCACAGATGGACAGAGCAGA					
ThuAla-mt-16	KM977798	205	F: CGACTGCCTTTGTCTGGTTT	(ga)11	NED			«neutral»
			R: CCACCAGTGAAGTACTGCTGAT					
ThuAla-mt-17	KM977799	226	F: GCTGCAGCTCATCTGTTCAC	(ac)11	6FAM			«neutral»
			R: TGGATTTCGTTTTCATTCTGTG					
ThuAla-mt-18	KM977800	110	F: TCTGCTCAAACCTGCTGACA	(tg)12	6FAM			«neutral»
			R: TACCGTCCCGATAAGAATGC					
ThuAla-Hki-01	KM977801	138	F: CTCACAGATGATGGGCAGG	(tc)12	NED	yes	Hexokinase type I	«non-neutral»
			R: TCCCTCCTCTGTGCATGTAA					
ThuAla-mt-19	KM977802	225	F: TCTGGACGTCTGATTGATCG	(ttc)12	NED			«neutral»
			R: GGCTGCCTTTTCTTGACAAC					
ThuAla-mt-20	KM977803	259	F: AGAACATGGGACCAGATTGC	(gt)12	VIC			«neutral»
			R: AGAATCGGTCAAAGGTCACG					
ThuAla-mt-21	KM977804	138	F: GTACCCTTCTCCCCTCAACC	(ca)13	PET			«neutral»
			R: CAATCTGCGTGAAGTGGGTA					
ThuAla-mt-22	KM977805	182	F: AACTTTGCTGCCAATCTGCT	(ac)13	6FAM			«neutral»
			R: GAATGCACGCTCATGTTCAC					
ThuAla-mt-23	KM977806	210	F: ATGATTTTAACCCTTGGCCC	(tg)13	PET			«neutral»
			R: CCAAATCACATCTGTGTCCG					
ThuAla-Und-03	KM977807	109	F: TGCGGGTTTTGTGAAATTCT	(ttc)14	PET	yes	Variant sequence and undeterminated gene	«non-neutral»
			R: ACTGTGGCAACCCCTAACAG					
ThuAla-mt-24	KM977808	118	F: GCTGGCAGTGCATATTCAAA	(ac)14	6FAM			«neutral»
			R: CAGTTGCAGCCTGTCATCAT					
ThuAla-mt-25	KM977809	132	F: GTCCCCAGTTGGACAAGATG	(ac)14	VIC			«neutral»
			R: CGCACAGCTGTTCCATTAAA					
ThuAla-mt-26	KM977810	151	F: TTCCCCTGCAGTGATTTAGG	(tg)14	PET			«neutral»
			R: AGGTACTGCCACTCCATTCG					
ThuAla-mt-27	KM977811	184	F: TCTGAAAGATAGACAGACATGCG	(ac)14	NED			«neutral»
			R: CAATTTTGCCAAAGCATCAA					
ThuAla-Tyr-01	KM977812	106	F: GAACATCAAGAACCACGAAGG	(gtt)15	VIC	yes	Receptor-type tyrosine-protein phosphatase-like N-like	«non-neutral»
			R: CCGTTCTCCCAGACCATCTA					
ThuAla-mt-28	KM977813	115	F: TGTCCCGGATACAGTTCTACA	(ac)15	NED			«neutral»
			R: CTGGCATGTTGATGTTGTCA					
ThuAla-mt-29	KM977814	116	F: ACAAATGCATTGCAGGTACG	(ac)15	PET			«neutral»
			R: CACCAGTGTGGCAACCATAA					
ThuAla-mt-30	KM977815	173	F: GCCAGCAGAGTATTCATCCC	(ca)15	6FAM	yes	Microsatellite	«neutral»
			R: ATTTAAGTAGGCGGCAGCAA					
ThuAla-Und-04	KM977816	137	F: CGAGGCATTATTTGATCCCTAT	(tg)16	VIC	yes	Gene undeterminated	«non-neutral»
			R: ACCTACAGGGAAGCCAGGAC					
ThuAla-mt-31	KM977817	106	F: TCATCATCTGGACAGATTGTGTAT	(tg)17	NED			«neutral»
			R: GAGGCAGAACATGAGGAAGG					
ThuAla-Und-05	KM977818	121	F: CAGTTCCTCCAAAGCAGGAG	(atg)17	NED	yes	Gene undeterminated	«non-neutral»
			R: AGAACAGGCAAAGATGCAGG					
ThuAla-Und-06	KM977819	138	F: AAGCAGCGTATTCCCAAAGA	(ag)17	6FAM	yes	Gene undeterminated	«non-neutral»
			R: GCCACTCGCCTGTTAACTTT					
ThuAla-mt-32	KM977820	142	F: TGCATGTTTGTTTGCAAGAG	(tg)17	PET			«neutral»
			R: GTGAGCTAAGTGCCACGACA					
ThuAla-mt-33	KM977821	225	F: GCTCCAAGTCCATCCTTGTC	(ac)18	PET			«neutral»
			R: GTAATGGGCTGACAGGTCGT					
ThuAla-mt-34	KM977822	236	F: CAGGCATGCAGAGGTAAACA	(ac)18	NED			«neutral»
			R: CAGCCTAATGAAGCCAGTGA					

Sequence range size in base pairs (complete sequence—primers, microsatellites and flanking region), primers sequence, number of repeats in the microsatellite motif determinate, microsatellite sequence corresponding at >75% alignment from GenBank NR and BOLD sequences, summary on the «non-neutral» gene alignment details, and information on the class of the markers («neutral», «non-neutral» and selected or not in the final panel).

PCR were performed in 25 μl reactions containing 5 ng of template DNA, 1X reaction buffer, 1.5 mM MgCl2, 0.24 mM dNTP, 0.1 μM of each primer, and 1U Taq polymerase. The PCR cycling consisted of an initial denaturation at 95°C for 10 min, followed by 40 cycles: denaturation at 95°C for 30 s, annealing at 55°C for 30 s, and extension at 72°C for 1 min and a final extension at 72°C for 10 min.

Out of the 95 markers, 70 markers were validated on agarose gel electrophoresis and 60 were selected for a polymorphism study (minimum of 3 alleles) on 15 albacore DNA samples (5 from area A, 5 from area B, and 5 from area C; Fig 1). PCR was performed following the same conditions as set above but with fluorescent forward primers (with 6’FAM, PET, VIC or NED fluorescent dye—Applied Biosystems). Each PCR amplicon was diluted with pure water (1:20), mixed with Hi-Di Formamide and GeneScan 500 LIZ dye size standard (Applied Biosystems), and were run on an Applied Biosystems 3730 XL DNA Analyzer. Alleles were scored using GeneMapper v 5.0 (Applied Biosystems). Of the 60 markers, we retained 43 markers based on technical (PCR feasibility and genotype reading) and molecular (optimal primer length of 20 bp (range 19–27 bp); optimal 50% GC content (range 25–60%); number of repeats greater than 8; most dinucleotide motif repeats; polymorphic (minimum of 4 alleles for each marker, observed on 15 individuals genotyped)) criteria.

Sequences similarities were sought by BLASTn (scanning databases of nucleotide collections with Megablast to search for highly similar sequences, [52] on the 43 markers. Sequences from GenBank NR and BOLD systems were downloaded for a local deployment (version 2014, GenBank; http://www.ncbi.nlm.nih.gov). We retained the alignment sequences with the expected value significance cut-off (E-value) ≤10–3. The degree of similarity was assessed using highly similar sequences (Megablast) and a ratio of similar bases (nucleotides) as a function of the microsatellite length to reveal the alignment sequences >75% (Table 2 and S2 Table). Sequence alignments were performed using the ClustalW program, setting parameters to default for gap criterions, followed by manual corrections with BioEdit software (http://www.ebi.ac.uk/Tools/msa/clustalo/).

Population diversity and structure analyses require random «neutral» microsatellite markers. 9 markers were detected as potentially encoded and 34 potentially «neutral» markers (Table 2). ThuAla-mt-30 has a high alignment and correspondence with a microsatellite sequence in *Cottus gobio*. The variability of 34 «neutral» microsatellites markers was screened using 136 individuals from the four areas. The level of diversity (allelic richness (Na); expected (He), expected unbiased from [53] (Hnb) and observed (Ho) heterozygosity) by locus was analyzed using GENETIX 4.05 [54]. Estimates of homozygote and heterozygote excess that differed significantly from zero (P<0.05) were calculated from the standard error in Pedant [55]. Probability of identity (PI) by locus was estimated using GenAlEx v6 [56]. PI is an advanced frequency-based analysis, also referred to as population match probability that provides an estimate of the average probability that two unrelated individuals will have the same multilocus genotype. It indicates the statistical power of marker loci. Deviations from Hardy-Weinberg equilibrium (HWE) were detected by exact tests and permutations (1 000 000 chains and 100 000 steps) and linkage disequilibrium by chi-square test and permutations (10 000) with ARLEQUIN version 3.1 [57]. Fisher’s inbreeding coefficient (Fis) and its significance was estimated by the exact test and Markov Chain method (10 000 dememorization, 1000 batches, 10 000 iterations per batch) using GENEPOP [58], and it was based on heterozygote excess to avoid disadvantages of common tests such as chi-square. Polymorphism Information Content (PIC) was generated in Cervus [59]. Null allele frequency (Fnull) was estimated with INEst [60] using the individual inbreeding model (estimates significantly different from zero, P<0.05), followed by MICRO-CHEKER [61] to understand the result of null alleles. Probability of parentage exclusion (PE1, single parent [62]); PE2, a second parent given a first parent assigned [63]; PE3, a pair of parents [62] was estimated per locus using INest. Assigning an individual determines the probability of assigning individuals to their likely population of origin. Genotyping error rate per allele, E1 referring to allelic dropout rate and E2 to the false allele rate, and the 95% confidence interval (CI), was evaluated using the number of repeated genotypes (Nrep and percentage (%) of the total number of individuals genotyped for each loci) and based on He computed in Pedant.

POWSIM software [64] was used to estimate the statistical power to detect levels of differentiation with a minimum of 30 individuals per area. Burn-in consisted of 1000 steps followed by 100 batches of 1000 steps. Chi-square and Fisher’s probabilities were used to test the significance of a Wright’s F-statistics (FST) value for each replicate run. The number of significant FST values in 1000 replicate simulations provided an estimate of statistical power for a given level of divergence, which was controlled by allowing frequencies to drift for a given number of generations.

Differentiation between the four areas (Fig 1) was visualized by Factorial Component Analysis in GENETIX with different numbers of markers. Global FST considering the 4 areas and the panel of potentially «neutral» microsatellite markers was estimated using GENETIX with 1000 bootstrap. Analysis of Molecular Variance (AMOVA) and Phi-statistics (analogous to F-statistics) were performed between the 4 areas using adegenet [65] and poppr [66] R package with 1 000 permutations.

SPOTG [67] was used to estimate the power of assignment of 4 populations, using 1000 runs. FST was equal to 0.005 and normal allele frequencies were used with the mean number of alleles equal to 17. The number of genetic markers to consider varied between 20 and 150 with 30 individuals. The number of individuals to sample varied between 30 and 500 with 34 markers. This software uses inputs from ARLEQUIN [68] and SIMCOAL [69].

The above analysis on the genetic diversity and structure were also applied to two «non-neutral» microsatellites markers in which the functions were well defined from GenBank NR and BOLD sequences alignment (ThuAla-Tcell-01, ThuAla-Hki-01; Table 1).

Finally, we tested the 34 selected microsatellites (Table 3) and 3 «non-neutral» markers (ThuAla-Tcell-01, ThuAla-Hki-01, and ThuAla-Tyr-01 –Table 2) on 4 species of Scombridae with high economic importance; three tuna species (*Thunnus albacares*, *Thunnus thynnus*, *Thunnus obesus*) and *Acanthocybium solandri*. PCR amplification was visualized in 2% agarose gel on 4 or 5 individuals per species.

**Table 3:** Summary statistics of the potentially selective «neutral» microsatellite markers (34) for albacore (*Thunnus alalunga*).

														Genotyping error rate	
Locus	Nind	Na	He	Hnb	Ho	PIC	Fnull	Fis	PI	PE1	PE2	PE3	Nrep (%)	E1 (CI 95%)	E2 (CI 95%)
ThuAla-mt-01	125	19	**0.92**	**0.92**	**0.72**	0.92	**0.098**	**0.22**	0.012	0.72	0.84	0.96	10(7)	0.00 (-0.00–0.22)	0.00 (0.00–0.09)
ThuAla-mt-02	134	27	0.91	0.92	0.90	0.91	0.014	0.02	0.013	0.71	0.83	0.95	11(8)	0.00 (-0.00–0.09)	0.00 (-0.00–0.07)
ThuAla-mt-03	121	27	**0.92**	**0.92**	**0.45**	0.91	**0.221**	**0.52**	0.013	0.71	0.83	0.95	12(9)	0.33 (-0.02–0.68)	0.00 (0.00–0.08)
ThuAla-mt-04	120	15	**0.90**	**0.91**	**0.68**	0.89	**0.109**	**0.25**	0.018	0.67	0.80	0.94	13(10)	0.00 (-0.00–0.42)	0.00 (0.00–0.06)
ThuAla-mt-05	132	8	0.59	0.59	0.55	0.54	0.046	**0.06**	0.217	0.19	0.35	0.53	13(10)	0.00 (0.00–0.21)	0.00 (0.00–0.06)
ThuAla-mt-06	136	10	0.70	0.70	0.71	0.66	0.014	0.00	0.136	0.29	0.46	0.65	13(10)	0.00 (0.00–0.07)	0.00 (0.00–0.06)
ThuAla-mt-07	135	12	0.69	0.69	0.66	0.64	0.019	0.04	0.143	0.28	0.45	0.64	13(10)	0.00 (0.00–0.07)	0.00 (0.00–0.06)
ThuAla-mt-08	135	14	**0.83**	**0.83**	**0.73**	0.81	0.054	**0.12**	0.049	0.50	0.67	0.85	13(10)	0.00 (-0.00–0.06)	0.00 (0.00–0.06)
ThuAla-mt-09	136	9	0.76	0.77	0.70	0.73	0.042	**0.09**	0.095	0.36	0.54	0.72	13(10)	0.00 (-0.00–0.06)	0.00 (0.00–0.06)
ThuAla-mt-10	136	27	0.87	0.87	0.85	0.85	0.014	**0.03**	0.030	0.59	0.74	0.90	13(10)	0.00 (-0.00–0.07)	0.00 (0.00–0.06)
ThuAla-mt-11	106	3	0.21	0.21	0.15	0.19	0.080	**0.27**	0.646	0.02	0.10	0.18	3(2)	0.00 (0.00–0.08)	0.00 (0.00–0.06)
ThuAla-mt-12	136	11	0.54	0.55	0.55	0.52	0.017	0.00	0.229	0.18	0.35	0.56	12(8)	0.00 (-0.00–0.13)	0.00 (-0.00–0.07)
ThuAla-mt-13	136	6	0.47	0.47	0.46	0.45	0.033	0.04	0.299	0.13	0.29	0.48	13(10)	0.00 (0.00–0.15)	0.00 (0.00–0.06)
ThuAla-mt-14	136	16	0.85	0.85	0.83	0.83	0.010	0.02	0.038	0.55	0.71	0.88	13(10)	0.00 (-0.00–0.07)	0.00 (-0.00–0.06)
ThuAla-mt-15	136	10	0.62	0.61	0.54	0.57	0.058	0.12	0.193	0.22	0.38	0.57	13(10)	0.00 (-0.00–0.11)	0.00 (0.00–0.06)
ThuAla-mt-16	134	20	**0.89**	**0.89**	**0.79**	0.88	0.051	0.12	0.021	0.65	0.79	0.93	12(9)	0.00 (0.00–0.07)	0.00 (0.00–0.06)
ThuAla-mt-17	51	10	**0.63**	**0.63**	**0.12**	0.60	**0.293**	**0.82**	0.166	0.24	0.43	0.63	6(4)	0.70 (0.03–1.81)	0.00 (-0.00–0.22)
ThuAla-mt-18	134	18	0.88	0.89	0.81	0.88	0.034	0.08	0.023	0.63	0.77	0.92	13(9)	0.00 (-0.00–0.06)	0.00 (0.00–0.06)
ThuAla-mt-19	115	13	**0.72**	**0.72**	**0.29**	0.68	**0.240**	**0.60**	0.121	0.32	0.50	0.69	11(8)	0.00 (0.00–0.14)	0.00 (0.00–0.07)
ThuAla-mt-20	130	36	**0.94**	**0.94**	**0.63**	0.94	**0.139**	**0.33**	0.007	0.78	0.88	0.97	13(9)	0.00 (-0.00–0.12)	0.00 (0.00–0.06)
ThuAla-mt-21	124	18	**0.86**	**0.87**	**0.45**	0.85	**0.207**	**0.48**	0.033	0.57	0.73	0.89	12(9)	0.42 (-0.02–0.78)	0.00 (0.00–0.09)
ThuAla-mt-22	125	20	**0.85**	**0.85**	**0.51**	0.83	**0.165**	**0.40**	0.038	0.55	0.71	0.88	11(8)	0.41 (-0.98–0.84)	0.00 (0.00–0.10)
ThuAla-mt-23	135	9	0.63	0.63	0.65	0.60	0.010	-0.03	0.166	0.24	0.42	0.63	13(9)	0.00 (0.00–0.22)	0.00 (0.00–0.06)
ThuAla-mt-24	136	13	0.85	0.85	0.84	0.83	0.014	0.01	0.041	0.53	0.70	0.87	13(10)	0.02 (0.00–0.06)	0.00 (0.00–0.06)
ThuAla-mt-25	136	13	0.77	0.77	0.79	0.74	0.012	-0.03	0.080	0.40	0.58	0.78	13(10)	0.00 (0.00–0.12)	0.00 (-0.00–0.07)
ThuAla-mt-26	136	16	0.69	0.70	0.67	0.65	0.019	0.04	0.140	0.29	0.46	0.65	13(10)	0.00 (-0.00–0.08)	0.00 (-0.00–0.06)
ThuAla-mt-27	121	20	**0.80**	**0.80**	**0.45**	0.79	**0.186**	**0.44**	0.052	0.48	0.65	0.86	9(6)	0.00 (-0.00–0.25)	0.00 (0.00–0.09)
ThuAla-mt-28	136	30	0.92	0.93	0.94	0.92	0.007	-0.02	0.011	0.74	0.85	0.96	13(10)	0.00 (0.00–0.06)	0.00 (-0.00–0.06)
ThuAla-mt-29	135	22	0.84	0.84	0.78	0.82	0.024	**0.06**	0.043	0.53	0.69	0.87	13(10)	0.00 (-0.00–0.06)	0.00 (-0.00–0.06)
ThuAla-mt-30	136	17	0.85	0.86	0.84	0.84	0.014	0.02	0.035	0.56	0.72	0.89	13(10)	0.00 (0.00–0.00)	0.00 (0.00–0.06)
ThuAla-mt-31	135	24	0.91	0.91	0.90	0.90	0.010	0.00	0.016	0.69	0.81	0.95	13(10)	0.00 (-0.00–0.07)	0.00 (-0.00–0.06)
ThuAla-mt-32	136	14	0.61	0.61	0.63	0.59	0.019	-0.02	0.172	0.23	0.42	0.64	13(10)	0.00 (-0.00–0.08)	0.00 (0.00–0.06)
ThuAla-mt-33	130	32	0.95	0.96	0.94	0.95	0.013	0.02	0.004	0.83	0.90	0.98	10(7)	0.00 (0.00–0.07)	0.00 (0.00–0.07)
ThuAla-mt-34	135	17	0.84	0.84	0.85	0.82	0.020	-0.01	0.042	0.53	0.69	0.87	13(10)	0.00 (-0.00–0.07)	0.00 (-0.00–0.06)
**Average**		16.94	0.77	0.77	0.66	0.75									

Number of individuals (Nind). Number of alleles (A). Expected (He), unbiased Nei's (1978) expected (H.n.b) and observed (HO) heterozygosity. Polymorphism information content (PIC). Null allele frequency (Fnull). Fisher’s inbreeding coefficient (Fis). Probability of identity (PI). Probability of exclusion (PE1, single parent; PE2, a second parent given a first parent assigned; PE3, a pair of parents). Number of repeated genotypes (Nrep and percentage (%) of the total number of individuals genotyped for each loci). Genotyping error rate per allele, E1 referring to allelic dropout rate and E2 to the false allele rate, and the 95% confidence interval (CI). Significant values are highlighted in bold (P<0.05) for heterozygote excess, Fnull, and Fis.

## Results

### Development of microsatellite panel on albacore tuna

**Fig 3:**
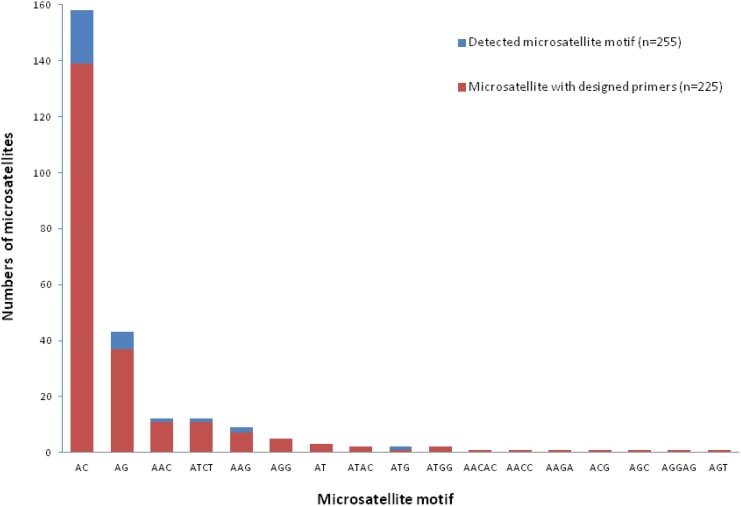
Number of microsatellites detected within good quality reads for primer design purpose and number of microsatellites with a successfully designed primer pair by motif type.

A total of 62 628 sequences with 4 285 (7%, Fig 2) unique and consensus sequences containing microsatellite markers were identified (motifs—type of repeat unit—range length of 248–288 bp) from 454 pyrosequencing. Genotyping profile characteristics of 1 670 primer pairs have been designed and described (S2 Table). Out of these sequences, 250 were high quality candidate microsatellite markers (Fig 3) and 225 were successfully designed. As expected, the most commonly found motifs were those used for library enrichment, in particular dinucleotide types AG and AC (37 and 139 microsatellites, respectively), followed by trinucleotides AAG, AAC, and AGG (7, 11, and 5 microsatellites, respectively) (Fig 3). However, although AT was not used as a motif for enrichment, 3 AT microsatellites were identified. Focusing on AG and AC motifs, the average number of repeated motifs was 8 for AG and 11 for AC with a maximum of 21 and 29, respectively (Fig 3). Allelic size range was 106 bp to 302 bp for 43 microsatellite markers (Table 2).

Among the 43 microsatellite markers, the BLASTn search revealed 9 microsatellites markers localized in a coding sequence. These 9 markers, called «non-neutral», have an E-value ≤ 10^−3^, except for marker ThuAla-Und-05 (Table 2 and S2 Table). Of the 9 «non-neutral» markers (>75% of alignment with a sequence mainly marine species, S2 Table), 6 undetermined function (ThuAla-Und-01, ThuAla-Und-02, ThuAla-Und-03, ThuAla-Und-04, ThuAla-Und-05, ThuAla-Und-06) presented some difficulties in the PCR process, in particular ThuAla-Und-01 and ThuAla-Und-03. These markers have not been included in the final panel. Concerning the remaining 3 «non-neutral» markers, ThuAla-Tcell-01 has a high ratio alignment (85%, S2 Table) with FERM and PDZ domain-containing protein 1-like. This domain is a protein often involved in localizing proteins to the plasma membrane and is both dispensable for the T cell receptor signal transduction [70] and could provide information on the immune system. ThuAla-Hki-01 has a high ratio alignment (80–83%, S2 Table) with the hexokinase type I which is one of the four hexokinases that participate in glycolysis playing a significant role in a wide range of cellular processes particularly in providing energy in muscle cells. ThuAla-Tyr-01 has a high ratio alignment (89%, S2 Table) with the receptor-type tyrosine-protein phosphatase-like N-like (PTPRN). It is an enzyme that regulates a variety of cellular processes (cell growth, differentiation, mitotic cycle, and oncogenic transformation) but the role in fish is unknown and it may have a general role in neuroendocrine functions, as in humans. In this study, we analyzed ThuAla-Hki-01 and ThuAla-Tcell-01 markers on overall albacore collected (136) as they are located to sequence having role in important biological traits (immunity and energy).

Genotyping was successfully performed on 136 albacore tunas collected from 4 different geographic areas (Tables 3 and 4) with the 34 supposed «neutral» and 2 «non-neutral» markers, ThuAla-Hki-01 and ThuAla-Tcell-01.

**Table 4:** Characteristics of two «non-neutral» microsatellite markers for albacore (*Thunnus alalunga*).

														Genotyping error rate	
Locus	Nind	Na	He	Hnb	Ho	PIC	Fnull	Fis	PI	PE1	PE2	PE3	Nrep (%)	E1 (CI 95%)	E2 (CI 95%)
ThuAla-Tcell-01	135	20	0.89	0.90	0.87	0.886	0.014	0.03	0.020	0.65	0.79	0.93	13(10)	0.00 (0.00–0.07)	0.00 (0.00–0.06)
ThuAla-Hki-01	136	9	0.27	0.27	0.24	0.254	0.041	**0.12**	0.551	0.04	0.14	0.26	13(10)	0.00 (-0.00–0.12)	0.00 (0.00–0.06)

Number of individuals (Nind). Number of alleles (A). Expected (He), unbiased Nei's (1978) expected (H.n.b) and observed (Ho) heterozygosity. Polymorphism information content (PIC). Null allele frequency (Fnull). Fisher’s inbreeding coefficient (Fis). Probability of identity (PI). Probability of exclusion (PE1, single parent; PE2, a second parent given a first parent assigned; PE3, a pair of parents). Number of repeated genotypes (Nrep and percentage (%) of the total number of individuals genotyped for each loci). Genotyping error rate per allele, E1 referring to allelic dropout rate and E2 to the false allele rate, and the 95% confidence interval (CI). Significant values are highlighted in bold (P<0.05) for heterozygote excess, Fnull, and Fis.

### “Encoding” markers analysis on albacore tuna

Number of alleles, heterozygosity and PIC was high for ThuAla-Tcell-01 and low for ThuAla-Hki-01 (Table 4). Both markers could be under balanced selection judging by the frequency of their allelic distribution, particularly ThuAla-Tcell-01 (S2 Fig), though these results are not sufficient to support this hypothesis. ThuAla-Tcell-01 presented low PI and high probability of parentage exclusion meaning high potential to assign individuals (Table 4). ThuAla-Hki-01 showed a significantly greater than zero estimate of Fis, a high PI and low probability of parentage exclusion (Table 4) and were detected in deviation from HWE. Concerning the linkage disequilibrium analysis, there is random association of alleles at all loci. These loci have a low genotyping error rate giving exactly repeatable genotypes with an observed error rate of 0.00 with low 95% CI.

### “Neutral” markers analysis on albacore tuna

Most of these markers had a large number of alleles per locus (A), ranging from 3 to 33 alleles (Table 3). 26 markers had at least 12 alleles and 10 markers had 16 or more alleles. The mean He and Ho varied, from 21% to 95% and from 15% to 94%, respectively. The PIC value averaged 0.75. Of all the markers, two presented low number of alleles, heterozygosity and PIC (ThuAla-mt-11, ThuAla-mt-13) (Table 3). 16 markers showed a significantly greater than zero estimate of Fis (Table 3) and they were detected in deviation from HWE. Null alleles may be present at 9 markers (ThuAla-mt-01, ThuAla-mt-03, ThuAla-mt-04, ThuAla-mt-17, ThuAla-mt-19, ThuAla-mt-20, ThuAla-mt-21, ThuAla-mt-22, and ThuAla-mt-27) (Table 3) as is also suggested by the significant excess of homozygotes (heterozygosity deficit). In these loci there was no evidence for scoring error due to stuttering and no evidence for large allele dropout. However, the significant null allele frequency in ThuAla-mt-03, ThuAla-mt-21, and ThuAla-mt-22 (Table 3) may be due to stuttering, resulting in possible scoring errors, as indicated by the highly significant shortage of heterozygote genotypes with alleles of one repeat unit difference. Concerning the linkage disequilibrium analysis, there is random association of alleles at all loci. Loci have a low genotyping error rate giving exactly repeatable genotypes with an observed error rate of 0.00 with low 95% CI except ThuAla-mt-03, ThuAla-mt-17, ThuAla-mt-21, and ThuAla-mt-22 (Table 3). These results confirm the stuttering for ThuAla-mt-03, ThuAla-mt-21, and ThuAla-mt-22. Concerning ThuAla-mt-17, this may be due to the null alleles.

The PI values ranged from 0.004 to 0.646 and the probability of exclusion from 0.02 to 0.98 on 34 microsatellite markers (Table 3). A total of 15 markers have a high PI (>0.05) (Table 3), meaning a high average probability that two unrelated individuals will have the same multilocus genotype (Table 3). It may be as a result of the low number of individuals (31–42) in the structure units.

### Comparison of selected panel with and without «non-neutral» markers

**Fig 4:**
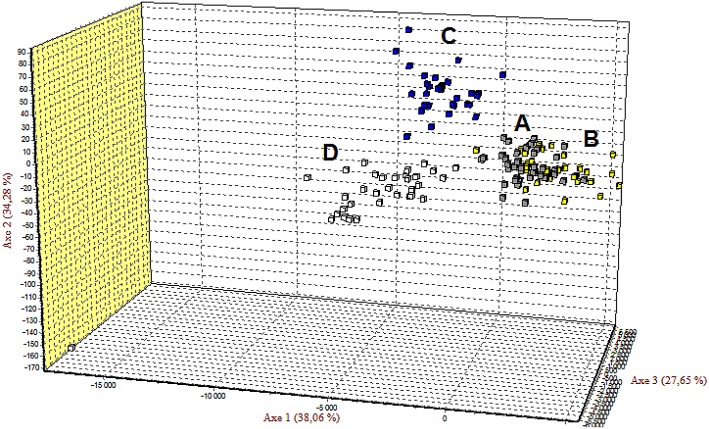
Factorial correspondence analysis (FCA) in three dimensions of four albacore populations. A (grey), B (yellow), C (blue), and D (white) (populations names as defined in Fig 1) with 36 markers (34 «neutral» and 2 «non-neutral» markers).

POWSIM simulations indicated that the 34 independent markers (34 «neutral») (Table 3) and 2 «non-neutral» markers (Table 4) were able to detect significant differences among samples with FST = 0.002 in around 90–95% of the tests and with FST = 0.005 in 100% of the tests (Table 5). Subsequently, the 34 high quality independent «neutral» markers were able to detect the same significant differences among samples with FST ≥ 0.002 in about 90–95% of the tests (Table 5). Finally, differentiation between the four areas was visualized by FCA with different numbers of markers (with and without “encoding markers” and potential Fnull markers (34–9 = 25 markers)) (Fig 4 and S3 Fig). The results obtained by power FST analysis and FCA analysis provided evidence of the suitability of 34 «neutral» microsatellite markers to determine the genetic relatedness among different populations and to evaluate their genetic variability. The addition of the two «non-neutral» markers does not improve or damage the analysis (Table 5, Fig 4 and S3 Fig). Jacknife by locus estimated the values of FST similar, around 0.0045 (standard deviation 0.00114) per markers. Global FST considering the 4 areas and the panel of potentially «neutral» microsatellite markers was low (0.005) with 95% CI equal to 0.003–0.007. FCA plots differentiated area C from D, whereas A and B were more similar (Fig 4).

**Table 5:** Probability of detecting a particular level of differentiation (FST) among populations of albacore with 1 000 replicates.

	36 markers(34 «neutral» + 2 «non-neutral»)		34 markers («neutral»)	
Fst	P Chi-squares	P Fisher	P Chi-squares	P Fisher
0.0005	0.17	0.20	0.16	0.19
0.001	0.49	0.49	0.46	0.47
0.002	0.95	0.91	0.94	0.90
0.005	1	1	1	1
Values > 0.005	1	1	1	1

However AMOVA analysis does not support this result, Phi-statistics was low (0.003) and not significant between C and D. The degree of differentiation between all area divisions was low and not significant, excepted weakly for A-D, and B-D, then highly between B and C (S4 Table). With regards to the high PI (15; Table 3), the number of individuals in each area may be insufficient, yet this probability may improve by increasing the number of markers providing high assignment discrimination. SPOTG estimated that with 30 individuals per sampling area, 40 microsatellites markers are the minimum number required to detect evolutionary and ecological processes with a power > 50% (S3 Table). SPOTG estimated that with 34 microsatellites, a minimum of 35 individuals from each sampling area is necessary to obtain a power > 50% and with 300 individuals the power increases to > 80% (S3 Table). SPOTG will not run simulations on more than 500 individuals.

### Panel tests on Scombridae species

The 34 «neutral» microsatellites markers at high quality (Table 3) and 2 «non-neutral» markers (Table 2) were amplified in 4 Scombridae species (*Thunnus albacares*, *Thunnus thynnus*, *Thunnus obesus*, *Acanthocybium solandri*). PCR amplification was successful for all markers in all *Thunnus* individuals tested (5) (Table 6). For *Acanthocybium solandri* the ratio of PCR amplification success was weak (1/4 individuals) with 4 markers (ThuAla-mt-03, ThuAla-mt-17, ThuAla-mt-19, and ThuAla-mt-20; Table 6). PCR amplification was weak with 10 markers in all 4 species (ThuAla-mt-14, ThuAla-mt-17, ThuAla-mt-26, ThuAla-mt-27, ThuAla-Tyr-01, ThuAla-mt-28, ThuAla-mt-29, ThuAla-mt-30, ThuAla-mt-31, ThuAla-mt-32; Table 6).

**Table 6:** PCR amplification results of 37 microsatellites markers tested on Scombridae species (*Thunnus albacares*, *Thunnus thynnus*, *Thunnus obesus*, *Acanthocybium solandri*) with 4 or 5 individuals per species.

Locus name	*Thunnus albacares*	*Acanthocybium solandri*	*Thunnus thynnus*	*Thunnus obesus*
ThuAla-mt-01	2/5	2/4	5/5	5/5
ThuAla-Tcell-01	5/5	4/4	5/5	3/5
ThuAla-mt-02	4/5	4/4	5/5	5/5
ThuAla-mt-03	4/5	**1/4**	5/5	1/5
ThuAla-mt-04	5/5	3/4	***4/5***	5/5
ThuAla-mt-05	**5/5**	4/4	5/5	5/5
ThuAla-mt-06	5/5	**4/4**	5/5	5/5
ThuAla-mt-07	***4/5***	***1/4***	***5/5***	***5/5***
ThuAla-mt-08	5/5	**3/4**	5/5	5/5
ThuAla-mt-09	5/5	4/4	5/5	5/5
ThuAla-mt-10	5/5	3/4	5/5	5/5
ThuAla-mt-11	5/5	4/4	5/5	2/5
ThuAla-mt-12	**5/5**	***2/4***	5/5	5/5
ThuAla-mt-13	5/5	4/4	5/5	5/5
ThuAla-mt-14	**5/5**	***3/4***	**5/5**	**5/5**
ThuAla-mt-15	5/5	**3/4**	5/5	**5/5**
ThuAla-mt-16	5/5	**4/4**	5/5	5/5
ThuAla-mt-17	**4/5**	**1/4**	***4/5***	**5/5**
ThuAla-mt-18	5/5	4/4	5/5	5/5
ThuAla-Hki-01	5/5	4/4	5/5	5/5
ThuAla-mt-19	5/5	**1/4**	5/5	5/5
ThuAla-mt-20	5/5	1/4	5/5	5/5
ThuAla-mt-21	5/5	**3/4**	5/5	5/5
ThuAla-mt-22	5/5	2/4	5/5	5/5
ThuAla-mt-23	5/5	4/4	5/5	5/5
ThuAla-mt-24	5/5	4/4	5/5	5/5
ThuAla-mt-25	5/5	***2/4***	5/5	5/5
ThuAla-mt-26	**5/5**	**4/4**	**5/5**	**5/5**
ThuAla-mt-27	**5/5**	**3/4**	**5/5**	**5/5**
ThuAla-Tyr-01	**5/5**	**4/4**	**5/5**	**5/5**
ThuAla-mt-28	**5/5**	***4/4***	**5/5**	**5/5**
ThuAla-mt-29	**5/5**	**4/4**	**5/5**	**5/5**
ThuAla-mt-30	**5/5**	**4/4**	**5/5**	**5/5**
ThuAla-mt-31	**5/5**	**4/4**	**5/5**	**5/5**
ThuAla-mt-32	**5/5**	**4/4**	**5/5**	**5/5**
ThuAla-mt-33	5/5	4/4	5/5	5/5
ThuAla-mt-34	5/5	4/4	5/5	5/5

Weak amplified product in bold. Very weak amplified product in bold and italic. Smear in grey.

## Discussion

The 454 GS FLX Titanium technology allowed fast development of polymorphic markers in albacore tuna, a non-model organism, for which low genomic information was available. This technology is interesting in term of cost and time and is effective in discovering high quality microsatellite markers for albacore tuna. This study provides the design of 1 670 microsatellite markers with all characteristics which could be used for different genetics projects on tuna (such as those carried by IOTC and ICCAT). Here, we chose a set of microsatellite markers, from the available markers designed, to investigate the albacore population genetics. Hence, the set of microsatellite markers developed in this study provides an additional tool to scientists who are investigating the genetic stock structure of this species and its implications for conservation and management measures. The same annealing temperature for optimal primer pairs allows easy multiplexing and faster manipulation at lower cost. Moreover, these markers display perfect microsatellite motif, making them easily usable in demographic inference, as in the coalescent theory [71, 29], which is a key question for albacore tuna (ex. population structure inferences’ implications on tuna species by [72]). Finally, most of 36 novel markers can also be used on other Scombridae species such as *Thunnus albacares*, *Thunnus thynnus*, *Thunnus obesus*, and *Acanthocybium solandri*.

The suitability of selected loci for population genetics analyses was assessed by computing several diversity and information content parameters and estimating 95% CI for genotyping error rate using repeated blind genotyping of the test panel. Analyses on the 136 individuals from all 4 areas results in a significant deviation from HWE. The 36 novel markers discovered constitute a useful tool for achieving detailed information on the genetic diversity and structure of this species and investigating its evolutionary history. Their high polymorphism, with the exception of 3 markers, proves their value in the characterization and evaluation of genetic diversity within and between populations.

Of the 9 «non-neutral» microsatellites markers discovered, two markers (ThuAla-Hki-01 and ThuAla-Tcell-01) were also characterized based on their link to sequence having potential role in a main biological trait (immunity and energy). Assessing statistical power by POWSIM confirmed that the panel of 25 «neutral» markers and of 34 «neutral» markers (Tables 2 and 3) could detect high levels of differentiation. However many markers have huge PI (15 from 34 «neutral»). In this study, FCA plots differentiated area C from D, whereas A and B were more similar. FCA did the best discrimination with all the markers. However AMOVA did not support this discrimination, particularly between C and D, and the Phi-statistics were low. Populations separated by lower genetic differentiation are less easy to make assignments, as is the case for albacore with a very low FST. The SPOTG simulations were made based on the mean number of alleles from this study. A higher number of individuals will increase the number of alleles and hence decrease the number of markers necessary to obtain an assignment power > 95%. The analysis by SPOTG revealed the necessity to increase the number of individuals and/or markers to detect evolutionary and ecological processes. Hence, we cannot conclude on the population genetics analysis due to the low number of individuals per sampling area. An increase in the number of individuals is required to assess the connectivity of albacore between geographic areas (Indian and Atlantic oceans).

Tests that produce different results based on increasing/decreasing the numbers of individuals used are encouraged to ensure the best individual assignment. Population structure and migration of albacore tuna is a challenging scientific question, but it is also a key question that needs to be addressed in terms of management of this species at the ocean-wide scale. There are at least six genetically distinct stocks of albacore, located in the North and South Pacific Ocean, North and South Atlantic Ocean, the Indian Ocean and the Mediterranean Sea [9, 42, 47, 73, 74]. Doubt subsists about the heterogeneity of stocks between the South Atlantic and Indian Oceans [14]. Small numbers of albacore may undertake inter-oceanic migrations between the South Atlantic Ocean and the Indian Ocean [75]. Nevertheless, the results are contrasted with one side genetic homogeneity [44] and the other heterogeneity [7, 76, 77]; between South Atlantic and Indian Oceans. The genetic studies, which did not detect any differentiation between populations, may not have enough resolution in the markers (type, polymorphism, and number) and/or the number of individuals sampled may have been too low.

A small number of «neutral» markers may not reflect inbreeding depression because they are unlikely to represent genome wide changes in homozygosity ([78] by [22]). Fine-scale genetic population structure often needs a large number of polymorphic microsatellite markers; and the final panel of microsatellite markers in this study corresponds to the general recommended number [27, 79, 80, 81]; under the condition of a minimum number of albacore individuals sampled. This panel could be expanded by existing markers (total 18) from literature on albacore population genetics studies [7, 42, 44, 47, 82].

## Supporting Information

S1 Fig Example of a typical electropherogram profile obtained with multiplexed PCR protocol for one individual and six microsatellite markers. (TIFF)

S2 Fig Allelic distribution of ThuAla-Tcell-01 microsatellite marker. (TIFF)

S3 Fig Factorial correspondence analysis (FCA) in three dimensions of four albacore populations. A (grey), B (yellow), C (blue), and D (white) (populations names as defined in Fig 1) with 36 markers (34 «neutral» and 2 «non-neutral» markers). (a) 34 «neutral» markers; (b) 27 (25 «neutral» and 2 «non-neutral» markers); (c) 25 «neutral» markers; and (d) microsatellite markers.
(TIFF)

S1 Table Selection criterion used to develop microsatellite markers. (XLS)

S2 Table Information on the alignment analysis corresponding to microsatellites (micro.) markers developed for albacore. 9 «non-neutral» markers and 1 marker (ThuAla-mt-30) align to *Cottus gobio* microsatellite corresponding sequence.
(XLSX)

S3 Table Assignment power with SPOTG simulations using different numbers of markers and individuals sampled. (XLSX)

S4 Table Matrix of pairwise Phi-statistics from AMOVA analysis. Lower matrice shows the Phi-statistics values of the four geographic location of albacore (A, B, C, and D). Significance was estimated using Monte Carlo tests and 1 000 permutations, * P-value<0.05, ** P-value <0.01, *** P-value <0.001.
(XLSX)

## References

[1] Collette B, Nauen C. 1983 Scombrids of the world—An annotated and illustrated catalogue of tunas, mackerels, bonitos and related species known to date. FAO Sp. Cat. 2, 137.

[2] Miyake MP, Miyabe N, Nakano H. 2004 Historical trends of tuna catches in the world. Rome: FAO Fisheries Technical Papers, 467, 74p.

[3] ISSF 2014. ISSF Tuna Stock Status Update, 2014: Status of the world fisheries for tuna. ISSF Technical Report 2014–09. International Seafood Sustainability Foundation, Washington, D.C., USA.

[4] IOTC—WPTmT05. 2014. Report of the Fifth session of the IOTC working party on temperate tunas. Indian Ocean Tuna Commission. IOTC–2014–WPTmT05–R[E].

[5] Collette BB, Carpenter KE, Polidoro BA. 2011 High value and long life-double jeopardy for tunas and billfishes. Science, 333, 291–292. 10.1126/science.1208730 21737699

[6] Juan-Jordá MJ, Mosqueira I, Cooper AB, Freire J, Dulvy NK. 2011 Global population trajectories of tunas and their relatives. Proceedings of the National Academy of Sciences of the United States of America, 108(51):20650–20655. 10.1073/pnas.1107743108 22143785 PMC3251139

[7] Albaina A, Iriondo M, Velado I, Laconcha U, Zarraonaindia I, Arrizabalaga H, et al 2013 Single nucleotide polymorphism discovery in albacore and Atlantic bluefin tuna provides insights into worldwide population structure. Animal Genetics, 44:678–692. 10.1111/age.12051 23668670

[8] Çiftci Y, Okumuş I. 2002 Fish Population Genetics and Application of Molecular Markers to Fisheries and Aquaculture—I: Basic Principles of Fish Population Genetics. Turkish Journal Fisheries and Aquatic Sciences, 2:145–155.

[9] Arrizabalaga H, Lopez-Rodas V, Costas E, González-Garcás A. 2007 Use of genetic data to assess the uncertainty in stock assessments due to the assumed stock structure: the case of albacore (*Thunnus alalunga*) from the Atlantic Ocean. Fisheries Bulletin, 105:140–6.

[10] Carlsson J, McDowell JR, Diaz-Jaimes P, Carlsson JEL, Boles SB, Gold JR, et al 2004 Microsatellite and mitochondrial DNA analyses of Atlantic bluefin tuna (*Thunnus thynnus thynnus*) population structure in the Mediterranean Sea. Molecular Ecology, 13:3345–3356. 15487994 10.1111/j.1365-294X.2004.02336.x

[11] Ely B, Vinas J, Alvarado Bremer JR, Black D, Lucas L, Covello K, et al 2005 Consequences of the historical demography on the global population structure of two highly migratory cosmopolitan marine fishes: the yellowfin tuna (*Thunnus albacares*) and the skipjack tuna (*Katsuwonus pelamis*). Evolutionary Biology 5:1–19. 15725349 10.1186/1471-2148-5-19 PMC554763

[12] Alvarado Bremer JR, Stequert B, Robertson NW, Ely B. 1998 Genetic evidence for interoceanic subdivision of bigeye tuna (*Thunnus obesus*) populations. Marine Biology 132, 547–557.

[13] Durand JD, Collet A, Chow S, Guinand B, Borsa P. 2005 Nuclear and mitochondrial DNA markers indicate unidirectional gene flow of Indo-Pacific to Atlantic bigeye tuna (*Thunnus obesus*) populations, and their admixture off southern Africa. Marine Biology 147:313–322.

[14] Nikolic N, Bourjea J. 2013. Differentiation of Albacore stock: review by oceanic regions. ICCAT Recueil de Documents Scientifiques. SCRS/2013/126.

[15] IOTC—SC15. 2012. Report of the Fifteenth Session of the IOTC Scientific Committee. Mahé, Seychelles, 10–15 December 2012. IOTC–2012–SC15–R[E].

[16] Ji P, Zhang Y, Li C, Zhao Z, Wang J, Li J, et al 2012 High throughput mining and characterization of microsatellites from common carp genome. International Journal of Molecular Sciences 13(8):9798–9807. 10.3390/ijms13089798 22949831 PMC3431829

[17] Iranawati F, Jung H, Chand V, Hurwood DA, Mather PB. 2012 Analysis of genome survey sequences and SSR marker development for Siamese mud carp, Henicorhynchus siamensis, using 454 pyrosequencing. International Journal of Molecular Sciences 13(9):10807–10827. 10.3390/ijms130910807 23109823 PMC3472715

[18] Wang J, Yu X, Zhao K, Zhang Y, Tong J, Peng Z. 2012 Microsatellite development for an endangered bream Megalobrama pellegrini (Teleostei, Cyprinidae) using 454 sequencing. International journal of Molecular Sciences, 13(3): 3009–3021. 10.3390/ijms13033009 22489139 PMC3317700

[19] Carlsson J, Gauthier DT, Carlsson JEL, Coughlan JP, Dillane E, Fitzgerald RD, et al 2013 Rapid, economical single-nucleotide polymorphism and microsatellite discovery based on *de novo* assembly of a reduced representation genome in a non-model organism: a case study of Atlantic cod *Gadus morhua* . Journal of fish biology 82(3):944–958. 10.1111/jfb.12034 23464553

[20] Nielsen EE, Hemmer-Hansen J, Larsen PF, Bekkevold D. 2009 Population genomics of marine fishes: identifying adaptative variation in space and time. Molecular Ecology, 18:3128–3150. 10.1111/j.1365-294X.2009.04272.x 19627488

[21] Waples RS, Naish KA. 2009 Genetic and evolutionary considerations in fishery management: Research needs for the future p 427–451 In: The future of fisheries science in North America. Beamish RJ and Rothschild BJ, editors. Springer, Dordrecht.

[22] Kirk H, Freeland JR. 2011 Applications and implications of «neutral» versus Non-»neutral» markers in molecular ecology. International Journal of Molecular Sciences, 12, 3966–3988. 10.3390/ijms12063966 21747718 PMC3131602

[23] Abdul-Muneer PM. 2014 Application of Microsatellite Markers in Conservation Genetics and Fisheries Management: Recent Advances in Population Structure Analysis and Conservation Strategies. Genetics Research International, ID 691759, 11 p. 10.1155/2014/691759 24808959 PMC3997932

[24] Okumuş I, Çiftci Y. 2003 Fish Population Genetics and Molecular Markers: II- Molecular Markers and Their Applications in Fisheries and Aquaculture. Turkish Journal of Fisheries and Aquatic Sciences, 3:51–79.

[25] Brooker AL, Cook D, Bentzen P, Wright JR, Doyle RW. 1994 Organization of microsatellites differs between mammals and cold-water teleost fishes. Canadian Journal of Fisheries and Aquatic Sciences, 51:1959–1966.

[26] O’Connell M, Wright JM. 1997 Microsatellite DNA in fishes. Reviews in *Fish Biology* and Fisheries, 7:331–363.

[27] Nikolic N, Butler J, Baglinière J-L, Laughton R, McMyn I.AG, Chevalet C. 2009 An examination of genetic diversity and effective population size in Atlantic salmon. Genetics Research, 91:1–18. 20122296 10.1017/S0016672309990346

[28] Oleksiak MF. 2010 Genomic approaches with natural fish populations. Journal of Fish Biology, 76:1067–1093. 10.1111/j.1095-8649.2010.02563.x 20409163 PMC3039449

[29] Nikolic N, Chevalet C. 2014 Detecting past changes of effective population size. Evolutionary Applications, (7):663–681. 25067949 10.1111/eva.12170 PMC4105917

[30] Nakamura I. 1985. FAO Species Catalogue. 5. Billfishes of the World. An Annotated and Illustrated Catalogue of Marlins, Sailfishes, Spearfishes and Swordfishes Known to Date. FAO Fisheries Synopsis, 125:65.

[31] Ward RD, Woodwark M, Skibinski DOF. 1994 A comparison of genetic diversity levels in marine, freshwater, and anadromous fishes. Journal of Fish Biology, 44:213–232.

[32] Hauser L, Carvalho GR. 2008 Paradigm shifts in marine fisheries genetics: ugly hypotheses slain by beautiful facts. Fish and Fisheries, 9:333–62.

[33] Yu HT, Lee YJ, Huang SW, Chiu TS. 2002 Genetic analysis of the populations of Japanese anchovy (Engraulidae: *Engraulis japonicus*) using microsatellite DNA. Marine Biotechnology (NY), 4(5):471–9. 10.1007/s10126-002-0035-8 14961240

[34] Bekkevold D, André C, Dahlgren TG, Clausen LA, Torstensen E, Mosegaard H, et al 2005 Environmental correlates of population differentiation in Atlantic herring. Evolution, 59(12):2656–68. 16526512

[35] Carlsson J, McDowell JR, Carlsson JEL, Olasdottir D. 2006 Genetic heterogeneity of Atlantic bluefin tuna caught in the eastern North Atlantic Ocean south of Iceland. ICES Journal of Marine Science, 63 (6):1111–1117.

[36] Gonzalez EG, Zardoya R. 2007 Relative role of life-history traits and historical factors in shaping genetic population structure of sardines (Sardina pilchardus). BMC Evolutionary Biology, 7:197 17953768 10.1186/1471-2148-7-197 PMC2204010

[37] Dos Santos SM, Klopper AW, Oosthuizen CJ, Bloomer P. 2008 Isolation and characterization of polymorphic tetranucleotide microsatellite loci in the pelagic perciform fish Pomatomus saltatrix (Linnaeus, 1766) from South Africa. Molecular Ecology Resources, 8(5):1065–7. 10.1111/j.1755-0998.2008.02156.x 21585973

[38] Gonzalez EG, Beerli P, Zardoya R. 2008 Genetic structuring and migration patterns of Atlantic bigeye tuna, Thunnus obesus (Lowe, 1839). BMC Evolutionary Biology, 8:252 10.1186/1471-2148-8-252 18798987 PMC2559848

[39] Was A, Gosling E, McCrann K, Mork J. 2008 Evidence for population structuring of blue whiting (*Micromesistius poutassou*) in the Northeast Atlantic. ICES Journal of Marine Science, 65:216–225.

[40] Gaggiotti OE, Bekkevold D, Jørgensen HB, Foll M, Carvalho GR, Andre C, et al 2009 Disentangling the effects of evolutionary, demographic, and environmental factors influencing genetic structure of natural populations: Atlantic herring as a case study. Evolution, 63(11):2939–51. 10.1111/j.1558-5646.2009.00779.x 19624724

[41] André C, Larsson LC, Laikre L, Bekkevold D, Brigham J, Carvalho GR, et al 2011 Detecting population structure in a high gene-flow species, Atlantic herring (*Clupea harengus*): direct, simultaneous evaluation of «neutral» vs. putatively selected loci. Heredity, 106, 270–280. 10.1038/hdy.2010.71 20551979 PMC3183876

[42] Davies CA, Gosling EM, Was A, Brophy D, Tysklind N. 2011 Microsatellite analysis of albacore tuna (*Thunnus alalunga*): population genetic structure in the North-East Atlantic Ocean and Mediterranean Sea. Marine Biology, 158: 2727–2740.

[43] Limborg MT, Hanel R, Debes PV, Ring AK, André C, Tsigenopoulos CS, et al 2012 Imprints from genetic drift and mutation imply relative divergence times across marine transition zones in a pan-European small pelagic fish (*Sprattus sprattu*s). Heredity (Edinb), 109(2):96–107. 22549515 10.1038/hdy.2012.18 PMC3400746

[44] Montes I, Iriondo M, Manzano C, Arrizabalaga H, Jimánez E, Pardo MA, et al 2012 Worldwide genetic structure of albacore (*Thunnus alalunga*) revealed by microsatellite DNA markers. Marine Ecology Progress Series, 471:183–191.

[45] Muths D, Le Couls S, Evano H, Grewe P, Bourjea J. 2013 Multi-genetic marker approach and spatio-temporal analysis suggest there is a single panmictic population of Swordfish *Xiphias gladius* in the Indian Ocean. Plos ONE, 8(5). 10.1371/journal.pone.0063558 PMC3661515 23717447

[46] Takagi M, Okamura T, Chow S, Taniguchi N. 1999 PCR primers for microsatellite loci in tuna species of the genus *Thunnus* and its application for population genetic study. Fisheries Sciences, 65:571–576.

[47] Takagi M, Okamura T, Chow S, Taniguchi N. 2001 Preliminary study of albacore (Thunnus alalunga) stock differentiation inferred from microsatellite DNA analysis. Fishery Bulletin, 99:697–701.

[48] Clark TB, Ma L, Saillant E, Gold JR. 2004 Microsatellite DNA markers for population-genetic studies of Atlantic bluefin tuna (*Thunnus thynnus thynnus*) and other species of genus Thunnus. Molecular Ecology Notes, 4:70–73.

[49] Malausa T, Gilles A, Meglecz E, Blanquart H, Duthoy S, Costedoat C, et al 2011 High-throughput microsatellite isolation through 454 GS-FLX Titanium pyrosequencing of enriched DNA libraries. Molecular Ecology Resources, 11:638–644. 10.1111/j.1755-0998.2011.02992.x 21676194

[50] Ward SM, Jasieniuk M. 2009 Review: sampling weedy and invasive plant populations for genetic diversity analysis. Weed Science, 57, 593–602.

[51] Meglécz E, Costedoat C, Dubut V, Gilles A, Malausa T, Pech N, et al 2010 QDD: a user-friendly program to select microsatellite markers and design primers from large sequencing projects. Bioinformatics, 26:403–404. 10.1093/bioinformatics/btp670 20007741

[52] Altschul SF, Madden TL, Schaffer AA, Zhang J, Zhang Z, Miller W, et al 1997 Gapped BLAST and PSI-BLAST: a new generation of protein database search programs. Nucleic Acids Research, 25:3389–3402. 9254694 10.1093/nar/25.17.3389 PMC146917

[53] Nei M. 1978 Estimation of average heterozygosity and genetic distance from a small number of individuals. Genetics, 89:583–590. 17248844 10.1093/genetics/89.3.583 PMC1213855

[54] Belkhir K, Borsa P, Chikhi L, Raufaste N, Bonhomme F. 1996 GENETIX, logiciel sous WindowsTM pour la génétique des populations Laboratoire Génome, Populations, Interactions CNRS UMR 5000. Montpellier: Université de Montpellier II.

[55] Johnson PCD, Haydon DT. 2007 Maximum-likelihood estimation of allelic dropout and false allele error rates from microsatellite genotypes in the absence of reference data. Genetics, 175:827–842. 17179070 10.1534/genetics.106.064618 PMC1800638

[56] Peakall R, Smouse P. 2006 GenAlEx 6: genetic analysis in Excel. Population genetic software for teaching and research. Molecular Ecology Notes, 6:288–295. 10.1093/bioinformatics/bts460 PMC3463245 22820204

[57] Excoffier L, Laval G, Schneider S. 2005 Arlequin ver. 3.0: An integrated software package for population genetics data analysis. Evolutionary Bioinformatics Online, 1:47–50. PMC2658868 19325852

[58] Raymond M, Rousset F. 1995 GENEPOP (version 1.2): population genetics software for exact tests and ecumenicism. Journal of Heredity, 86:248–249.

[59] Kalinowski ST, Taper ML, Marshall TC. 2007 Revising how the computer program CERVUS accommodates genotyping error increases success in paternity assignment. Molecular Ecology, 16:1099–1106. 17305863 10.1111/j.1365-294X.2007.03089.x

[60] Chybicki IJ, Burczyk J. 2009 Simultaneous estimation of null alleles and inbreeding coefficients. Journal of Heredity, 100:106–113. 10.1093/jhered/esn088 18936113

[61] Oosterhout CV, Hutchinson WF, Wills DPM, Shipley P. 2004 Micro-checker: software for identifying and correcting genotyping errors in microsatellite data. Molecular Ecology Notes, 4:535–538.

[62] Jamieson A, Taylor SCS. 1997 Comparisons of three probability formulae for parentage exclusion. Animal Genetics 28, 397–400. 9616104 10.1111/j.1365-2052.1997.00186.x

[63] Jamieson A. 1994 The effectiveness of using co-dominant polymorphic allelic series for (1) checking pedigrees and (2) distinguishing full-sib pair members. Animal Genetics 25 (Suppl. 1):37–44. 7943982 10.1111/j.1365-2052.1994.tb00401.x

[64] Ryman N, Palm S, Andrá C, Carvalho GR, Dahlgren TG, Jorde PE, et al 2006 Power for detecting genetic divergence: differences between statistical methods and marker loci. Molecular Ecology 15, 2031–45. 16780422 10.1111/j.1365-294X.2006.02839.x

[65] Jombart T. 2008 adegenet: a R package for the multivariate analysis of genetic markers Bioinformatics 24: 1403–1405. 10.1093/bioinformatics/btn129 18397895

[66] Kamvar ZN, Tabima JF, Grünwald NJ. 2014 Poppr: an R package for genetic analysis of populations with clonal, partially clonal, and/or sexual reproduction. PeerJ 2:e281 10.7717/peerj.281 24688859 PMC3961149

[67] Hoban S, Gaggiotti O, Bertorelle G. 2013 Sample Planning Optimization Tool for conservation and population Genetics (SPOTG): a software for choosing the appropriate number of markers and samples. Methods in Ecology and Evolution, 4: 299–303.

[68] Excoffier L, Lischer HEL. 2010 Arlequin suite ver 3.5: A new series of programs to perform population genetics analyses under Linux and Windows. Molecular Ecology Resources. 10: 564–567. 10.1111/j.1755-0998.2010.02847.x 21565059

[69] Laval G, Excoffier L. 2004 SIMCOAL 2.0: a program to simulate genomic diversity over large recombining regions in a subdivided population with a complex history. Bioinformatics 20(15): 2485–2487 15117750 10.1093/bioinformatics/bth264

[70] Bauler TJ, Hendriks WJAJ, King PD. 2008 The FERM and PDZ Domain-Containing Protein Tyrosine Phosphatases, PTPN4 and PTPN3, Are Both Dispensable for T Cell Receptor Signal Transduction. Plos One, 3:12. 10.1371/journal.pone.0004014 PMC2602985 19107198

[71] Chevalet C, Nikolic N. 2010 Distribution of coalescent times and distances between microsatellite alleles with changing effective population size. Theoretical Population Biology, 77:152–163. 10.1016/j.tpb.2010.01.001 20085779

[72] Kolody D, Grewe P, Davies C, Proctor C. 2013. Are Indian Ocean tuna populations assessed and managed at the appropriate spatial scale? A brief review of the evidence and implications. IOTC-2013-WPTT15-13.

[73] Viñas J, Alvarado Bremer JR, Pla C. 2004 Inter-oceanic genetic differentiation among albacore (*Thunnus alalunga*) populations. Marine Biology, 145:225–232.

[74] Chow S, Ushiama H. 1995 Global population structure of albacore (*Thunnus alalunga*) inferred by RFLP analysis of the mitochondrial ATPase gene. Marine Biology, 123:39–45.

[75] Beardsley GL. 1969 Proposed Migrations of Albacore, *Thunnus alalunga*, in the Atlantic Ocean. Transactions of the American Fisheries Society, 98(4):589–598.

[76] Yeh SY, Treng TD, Hui CF, Penney AJ. 1997 Mitochondrial DNA sequence analysis on Albacore *Thunnus alalunga*, meat samples collected from the waters off western South Africa and the Eastern Indian Ocean. ICCAT, Scientific Papper, 46:152–159.

[77] Laconcha U, Iriondo M, Arrizabalaga H, Manzano C, Markaide P, Montes I, et al 2015 New Nuclear SNP Markers Unravel the Genetic Structure and Effective Population Size of Albacore Tuna (*Thunnus alalunga*). PLoS ONE 10(6): e0128247 10.1371/journal.pone.0128247 26090851 PMC4474438

[78] Chapman JR, Nakagawa S, Coltman DW, Slate J, Sheldon BC. 2009 A quantitative review of heterozygosity-fitness correlations in animal populations. Molecular Ecology, 18, 2746–2765. 10.1111/j.1365-294X.2009.04247.x 19500255

[79] Barker JSF, Bradley DG, Fries R, Hill WG, Nei M, Wayne RK. 1993. An integrated global program to establish the genetic relationships among the breeds of each domestic animal species. Rome: FAO Animal Production and Health Paper, Report of a working group.

[80] Luikart G, Cornuet J. 1998 Empirical evaluation of a test for identifying recently bottlenecked populations from allele frequency data. Conservation Biology, 12, 228–237.

[81] SanCristobal M, Chevalet C, Haley CS, Joosten R, Rattink AP, Harlizius B, et al 2006 Genetic diversity within and between European pig breeds using microsatellite markers. Animal Genetics, 37:189–198. 16734675 10.1111/j.1365-2052.2005.01385.x

[82] Nakadate M, Viñas J, Corriero A, Clarke S, Suzuki N, Chow S. 2005 Genetic isolation between Atlantic and Mediterranean albacore populations inferred from mitochondrial and nuclear DNA markers. Journal of Fish Biology, 66:1545–57.

