# Supplementary figures and images for: Discovery of Genome-Wide Microsatellite Markers in Scombridae: A Pilot Study on Albacore Tuna
Source: PLoS One. 2015 Nov 6;10(11):e0141830. doi: 10.1371/journal.pone.0141830 (PMC4636268; doi:10.1371/journal.pone.0141830)

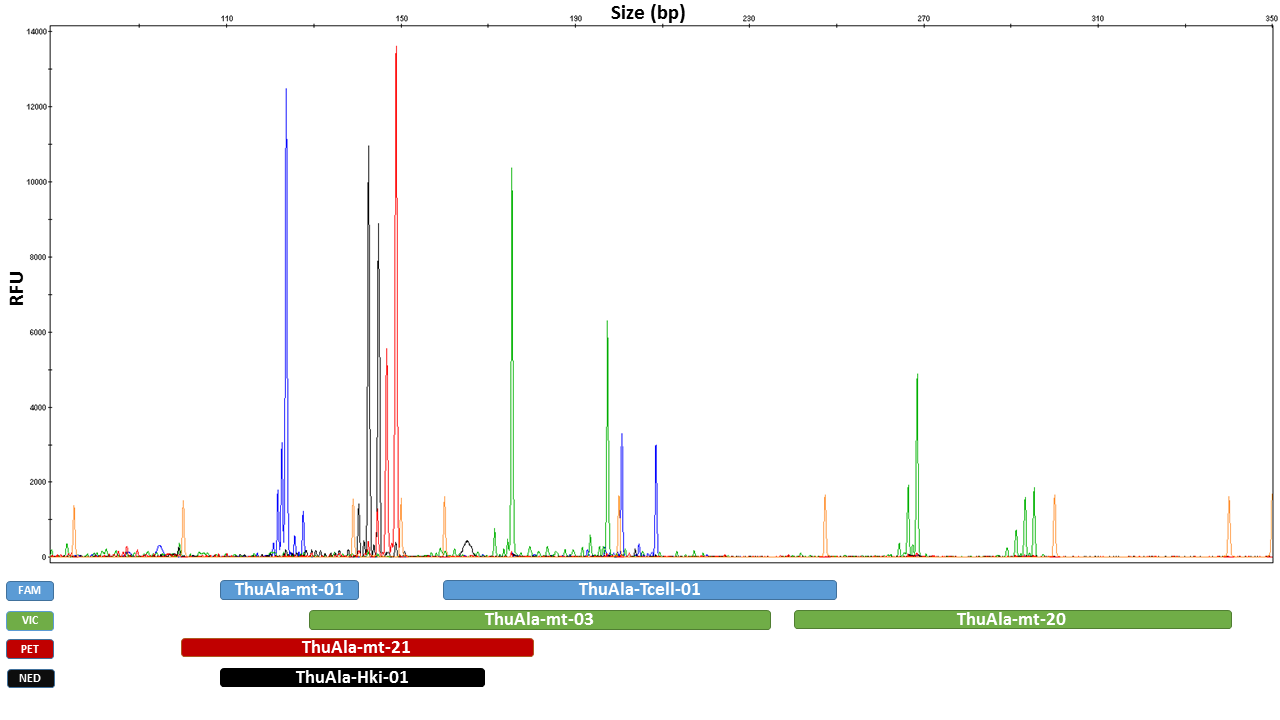

Supplement: S1 Fig — (TIFF) [file pone.0141830.s001.tiff]

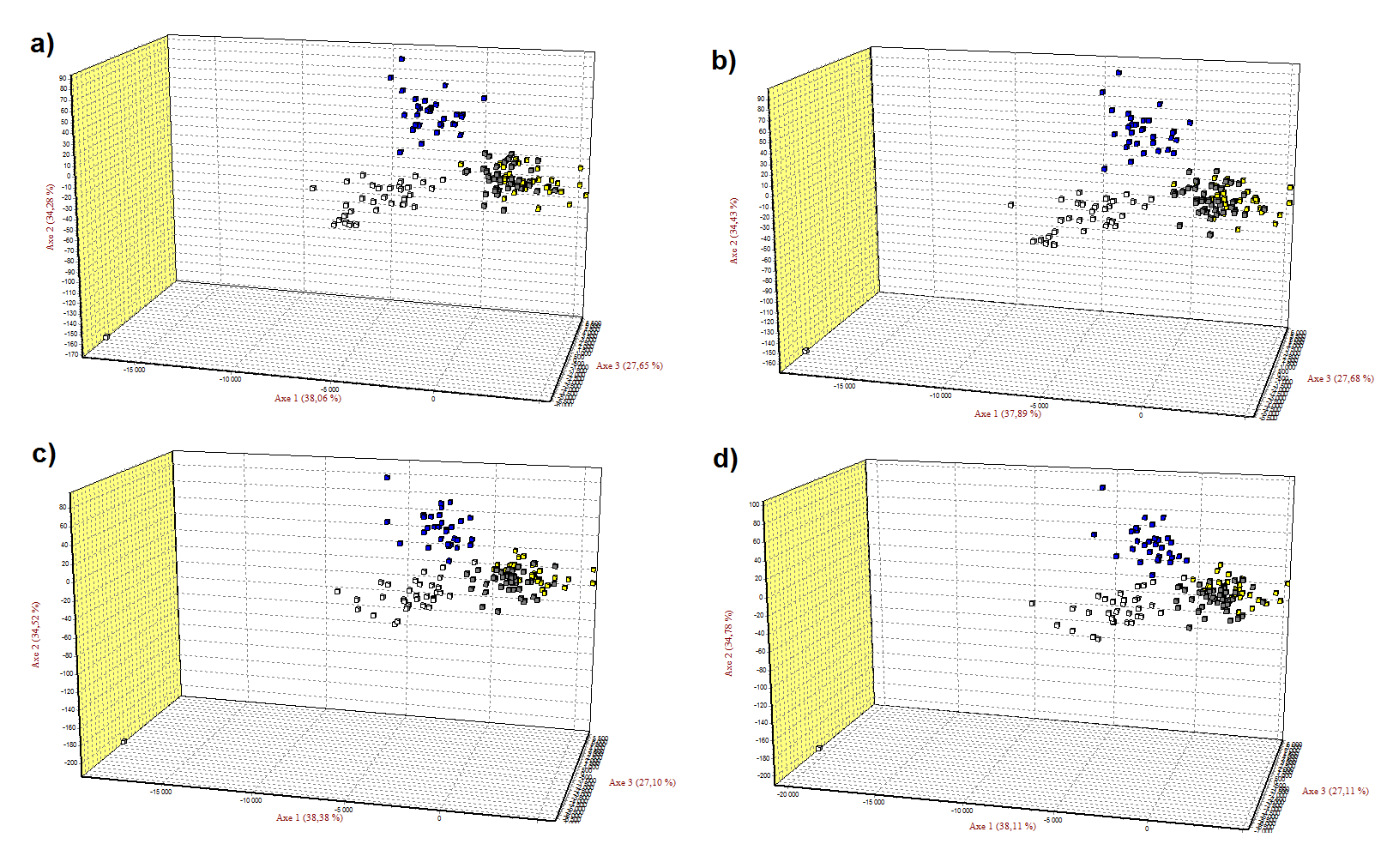

Supplement: S3 Fig — A (grey), B (yellow), C (blue), and D (white) (populations names as defined in Fig 1) with 36 markers (34 «neutral» and 2 «non-neutral» markers). (a) 34 «neutral» markers; (b) 27 (25 «neutral» and 2 «non-neutral» markers); (c) 25 «neutral» markers; and (d) microsatellite markers. (TIFF) [file pone.0141830.s003.tiff]
